# Percutaneous transluminal stenting for superior vena cava syndrome caused by malignant tumors: a single-center retrospective study

**DOI:** 10.1186/s13019-021-01418-w

**Published:** 2021-03-20

**Authors:** Haitao Liu, Yahua Li, Yang Wang, Lei Yan, Pengli Zhou, Xinwei Han

**Affiliations:** 1 Department of Interventional Radiology, The First Affiliated Hospital of Zhengzhou University, Zhengzhou, China; 2 Interventional Treatment and Clinical Research Center of Henan Province, Zhengzhou, China

**Keywords:** Tumor, Superior vena cava syndrome, Angiography, Stent

## Abstract

**Objectives:**

To evaluate the efficacy of percutaneous stent placement in the treatment of superior vena cava syndrome caused by malignant tumors.

**Methods:**

We retrospectively analyzed the clinical data of 32 patients with superior vena cava syndrome who underwent percutaneous endovascular stent treatment in our department from 2015 to 2019 due to malignant tumors and summarized the patient’s sex, age, tumor type, endovascular treatment plan, complications and postoperative follow-up.

**Results:**

All patients successfully underwent percutaneous intraluminal stent placement with digital subtraction angiography (DSA). Thirty-seven endovascular stents were implanted in 32 patients, including 21 Eluminexx stents, 12 Wallstent stents and 4 covered stents. The technical success rate was 100%, and there were no serious surgery-related complications. The remission rate of clinical symptoms was 53.1% (17/32) at 24 h and 84.4% (27/32) at 48 h. After 48 h, the symptoms of the remaining patients were slowly relieved, and the symptom relief rate was 100% at 7 days. The follow-up period was 1.5–24 months, with an average follow-up period of 6.5 months. During the follow-up, 3 patients had restenosis and 1 patient had secondary thrombosis in the stent. Their symptoms were relieved after the second treatment.

**Conclusion:**

For superior vena cava syndrome caused by malignant tumors, percutaneous endoluminal stent therapy can quickly and effectively relieve the clinical symptoms of patients, and the incidence of complications is low.

## Background

Superior vena cava syndrome (SVCS) can be secondary to exogenous compression, central venous catheter thrombosis, upper limb arteriovenous fistula, infection or fibrosis after radiotherapy, etc. [1]. The most common cause is exogenous compression by malignant tumors or invasion of bronchial lung cancer [2]. The main symptoms of SVCS are wheezing, dysphagia, swelling, and venous dilatation in the head and upper body, and some cases can cause brain edema, which may lead to nervous system-related symptoms [3, 4].

SVCS caused by malignant tumors mainly actively treats the primary disease and restores the blood flow of the superior vena cava as soon as possible to relieve clinical symptoms. Palliative treatment is the most suitable option for such patients. Maurizi and Sato et al. showed that it is feasible to perform superior vena cava resection for patients with malignant tumors, but complications such as empyema, bronchial fistula, and superior vena cava reocclusion also occurred [5, 6]. In the past, it was thought that tumor compression was the main cause of superior vena cava syndrome caused by tumors. Hinton et al. reported a case of a non-small cell lung cancer patient, who received chemotherapy, and the tumor was reduced by more than 50%, but the superior vena cava stenosis was not relieved [7]. Lanciego recommends Wallstent endoprosthesis as the first choice for palliative treatment of superior vena cava syndrome [8]. In this study, we retrospectively analyzed the case data of patients with malignant superior vena cava obstruction who underwent stent placement in our hospital to verify its effectiveness, safety and efficacy.

## Methods

### Patient selection

We retrospectively analyzed the data of 32 patients with superior vena cava syndrome who underwent percutaneous endovascular stenting in the First Affiliated Hospital of Zhengzhou University from 2015 to 2019, including the general information of the patient, the primary disease, the surgical indications, the surgical status, postoperative efficacy, complications and follow-up. The general characteristics of the patients are shown in Table 1. All included patients had malignant tumors confirmed by pathology, including lung cancer, esophageal cancer, lymphoma, thymoma and mediastinal metastasis. This study was approved by the Ethical Review Committee of the First Affiliated Hospital of Zhengzhou University. All biological samples and images were obtained after the patient’s written informed consent.

**Table 1:** General information of patients

Characteristic	Value
Age (years)	57 ± 12.3 (29–80)
Sex	
Male	25
Female	7
Causes of SVC syndrome	
Lung cancer	21
Esophageal cancer	5
Lymphoma	3
Mediastinal metastases	1
Thymoma	2
Stenosis	
Superior vena cava stenosis	19
Superior vena cava occlusion	6
Superior vena cava + brachiocephalic vein stenosis	7
Preoperative ECOG score	
0	3
1	7
2	19
3	3
4	0
5	0

*ECOG* performance status made by Eastern Cooperative Oncology Group

### Interventional therapy

Before the operation, routine blood tests, coagulation function tests, and blood and urine biochemical tests were completed, and the superior vena cava vessels were reconstructed by chest computed tomography angiography to evaluate the cause, location, range and degree of superior vena cava stenosis and whether it was complicated with thrombosis.

The DSA instrument used during the operation was Siemens Artis Zeego, Germany, and the parameters of the high-pressure syringe were set to flow rate 8 ml/s, flow rate 20 ml, and pressure 300 psi. All operations were performed under local anesthesia. Venous access was established first, and then an electrocardiographic monitor was connected. The bilateral inguinal area was disinfected and covered with towels, and 1% lidocaine was used for local anesthesia. After the right femoral vein was punctured with an 18G puncture needle using the modified Seldinger method, a 0.035-in. hydrophilic guide wire and 5F catheter were introduced (if necessary, a long sheath tube could be inserted first, and then a guide wire and catheter could be introduced through the sheath). After entering the right subclavian vein throughthe stenosis and occlusion, superior vena cava angiography was performed to determine the lesion length, stenosis degree, blood flow velocity, normal vessel diameter and collateral circulation formation of the superior vena cava. When the superior vena cava was completely occluded, catheter and guide wires were used to open the occluded part bluntly or sharply.

After confirming the position of the occlusion by angiography, the intravascular stent was placed. Single stent placement: the stent was introduced along the stiffened guide wire to cross the lesion, and the two ends protruded approximately 1 ~ 2 cm into the normal lumen. After the positioning was confirmed to be accurate, it was released. After the stent was placed, the angiography was reviewed to confirm the stent position, stent deployment, stenosis recovery and blood flow through. If the stent expanded poorly, a balloon dilatation catheter was introduced to expand the poorly expanded area. “Y” - shaped double stent placement: after the guide wire and catheter were connected to the normal subclavian vein, the stiff guide wire and stent delivery system was introduced. After confirming the position is accurate, the stent was released in one brachiocephalic vein. Then, the guide wire was placed in the other brachiocephalic vein through the mesh of the stent, and the stiff guide wire and balloon catheter were introduced to expand the mesh of the stent and the narrow segment of the brachiocephalic vein. Then, another stent was introduced through the mesh of the previous stent, the distal end of the stent being placed in the normal blood vessel, and the proximal end being placed in the previously released stent.

Angiography was performed again to confirm whether the blood flow in the stent was smooth, whether there was residual stenosis, and whether the peripheral collateral vein development was reduced. If necessary, a balloon was used to expand the stent connection. After the operation, low molecular weight heparin was injected subcutaneously at 4000 U/12 h, and warfarin was administered orally for anticoagulation treatment. During anticoagulation treatment, coagulation function was monitored, and warfarin use was adjusted according to the INR (international normalized ratio). When the warfarin took effect, low molecular weight heparin administration was stopped.

## Results

**Fig. 1:**
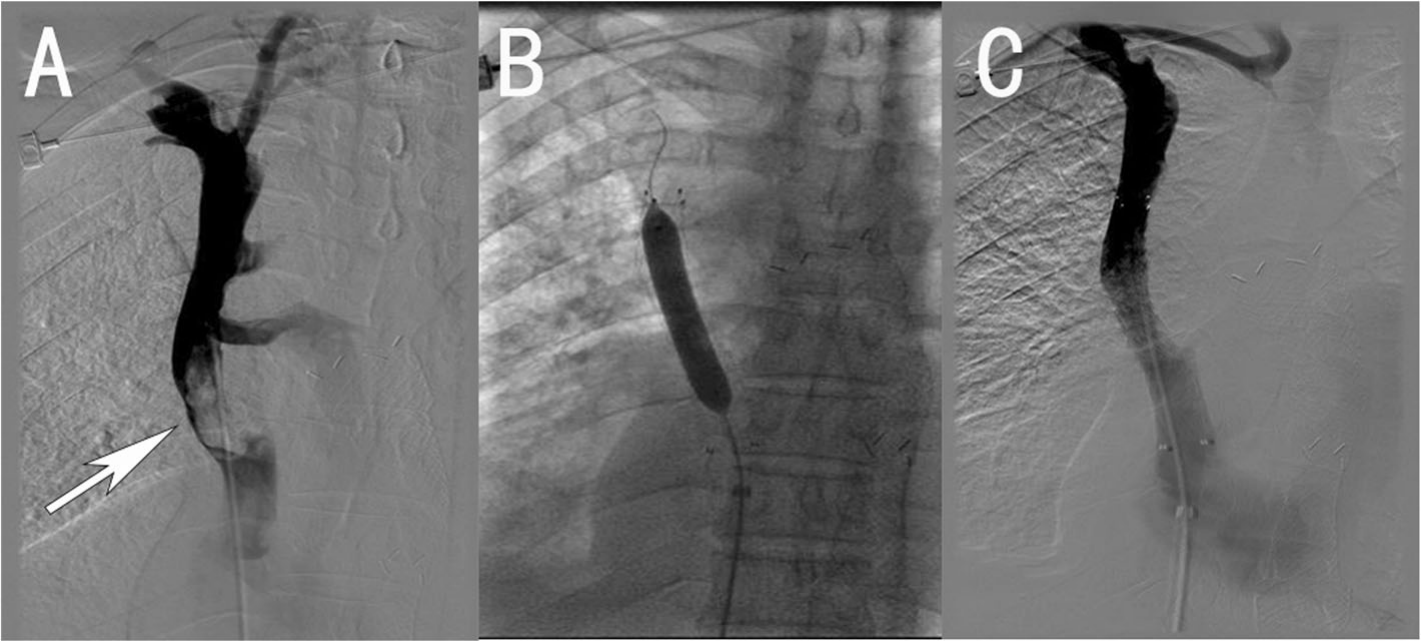
A patient with superior vena cava syndrome caused by lung cancer. **a**. Angiogram of the patient’s superior vena cava. **b**. Balloon expansion after the stent is placed. **c**. Angiographic image after treatment showing that the superior vena cava is unobstructed

**Fig. 2:**
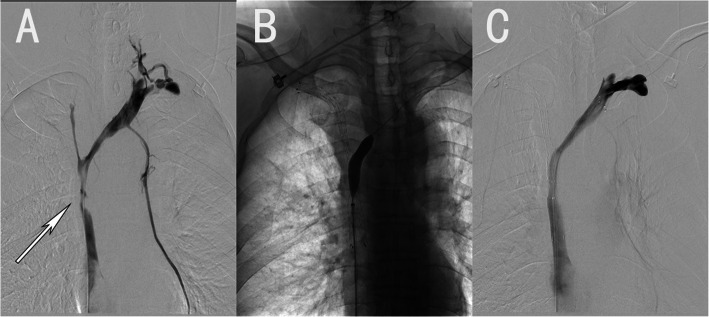
A patient with bilateral brachiocephalic vein and superior vena cava compression caused by lung cancer. **a**. The patient’s superior vena cava image showing filling defectsin the left brachiocephalic vein, right brachiocephalic vein and superior vena cava. **b**. The process of balloon expansion of the superior vena cava stent mesh. **c**. The bilateral brachiocephalic vein and superior vena cava after implantation (2 stents)

In this study, 32 patients successfully completed percutaneous transluminal stent implantation with a technical success rate of 100%. Most patients (29 cases) underwent the right femoral vein approach, 2 patients underwent the right subclavian vein approach, and 1 patient underwent the right femoral vein approach combined with the right basilica vein two-way approach. Sixteen patients only underwent stent implantation, 15 patients underwent stent implantation combined with balloon dilation (Fig. 1), and 1 patient underwent stent implantation after thrombus aspiration. Two stents were implanted in 5 patients, 2 of which were covered stents combined with bare metal stents, while the other 2 patients were treated with the stent drilling mesh technique, called the “Y” release, taking into account the superior vena cava and brachiocephalic vein (Fig. 2). A total of 37 stents were used, including 21 E·Luminexx Vascular Stents (BardGmbH/Angiomed, Karlsruhe, Germany), 12 Wallstent stents (Boston Scientific Corporation, MA, USA) and 4 Fluency Plus Vascular Stent Grafts (Bard GmbH/Angiomed, Karlsruhe, Germany). The length of the stents ranged from 4 cm to 12 cm, and their diameter ranged from 12 mm to 22 mm.

The clinical symptom relief rate at 24 h postoperation was 53.1% (17/32), and the clinical symptom relief rate at 48 h was 84.4% (27/32). After 48 h, the symptoms of the remaining patients were relieved slowly, and the symptom relief rate was 100% at 7 days. One patient had low back pain, and one patient had right upper limb and shoulder pain. After symptomatic treatment, the pain gradually disappeared after 3–5 days.

**Fig. 3:**
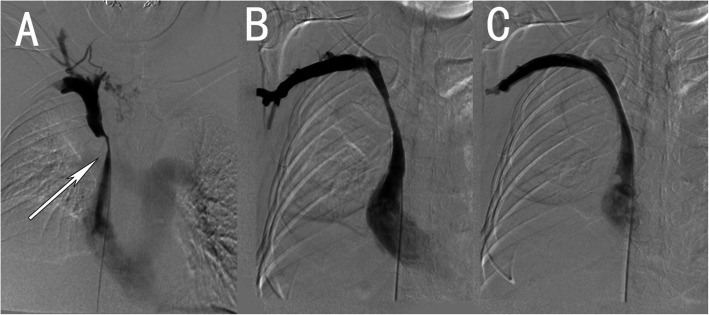
A case of superior vena cava syndrome with restenosis after stenting. **a**. DSA image showing obvious compression and stenosis of the superior vena cava. **b**. Angiographic image 16 months after stent implantation showing a filling defect in the superior vena cava stent. **c**. Placement of a longer stent in the original stent

**Fig. 4:**
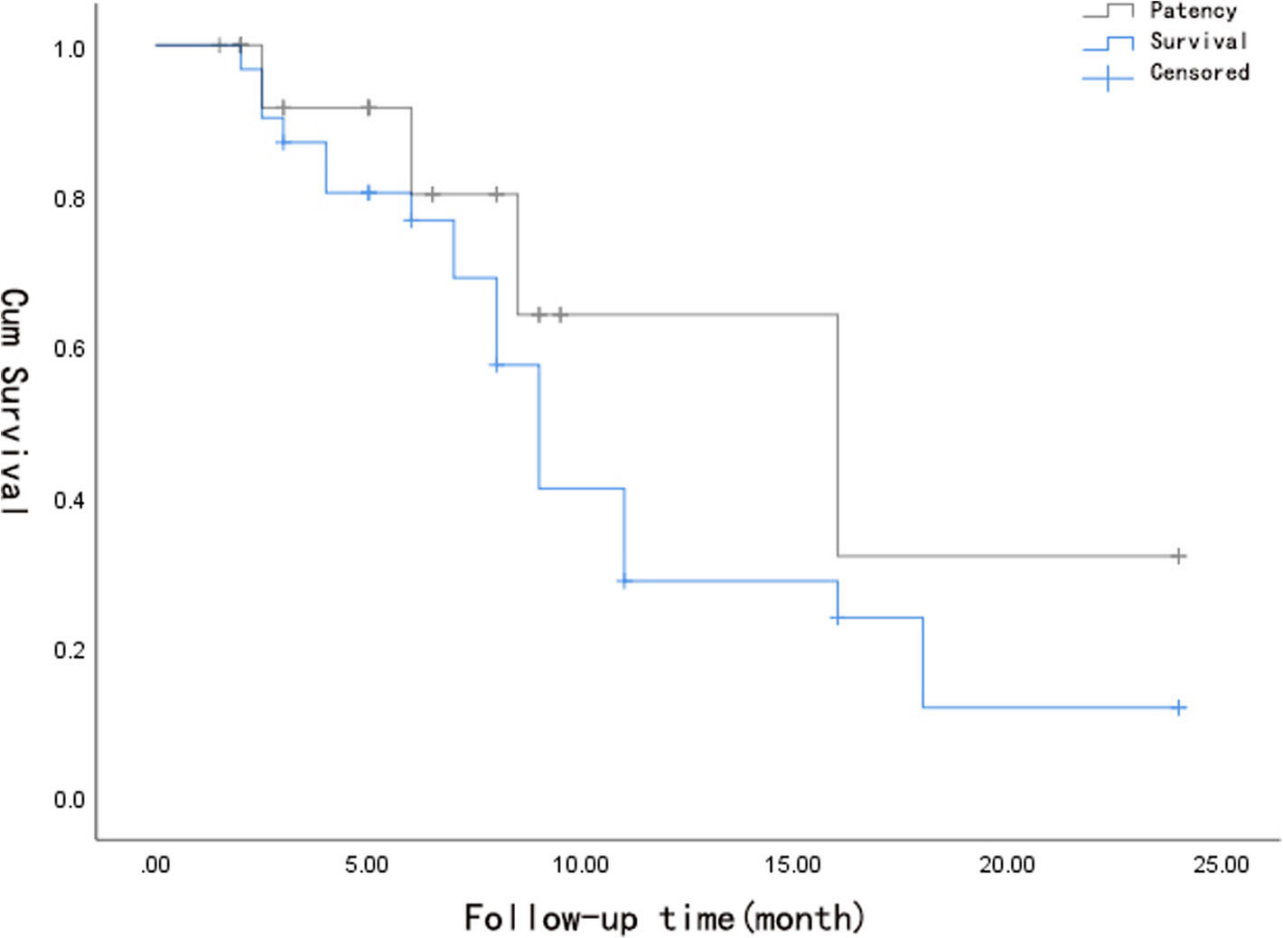
Kaplan-Meier stent patency and survival curve

Postoperative follow-up: the follow-up time was 1.5–24 months, with an average of 6.5 months. Symptoms recurred in 4 patients at 2.5, 6, 8.5 and 16 months. After stent implantation in 3 patients, the stenosis was relieved (Fig. 3); 1 patient underwent balloon dilation and catheter thrombolysis, and the symptoms improved 3 days after thrombolysis. The patency rates of the stents were 91.7, 80.2 and 64.2% at 3, 6 and 12 months postoperation, respectively. The stent patency and survival time curve is shown in Fig. 4.

## Discussion

Superior vena cava syndrome often occurs in malignant diseases, especially bronchial cancer, lymphoma and metastatic tumors [9]. Reconstruction of the superior vena cava and bypass grafting can be used as adjuvant treatments for malignant superior vena cava syndrome. However, due to the extensive trauma, the high risk of anesthesia, and the general inability of patients to tolerate the surgery, it hasrarely been used [10, 11]. At present, medical radiotherapy and chemotherapy are still the main treatment options for patients with tumors combined with superior vena cava syndrome because they can treat the primary tumor, reduce the lesion area and reduce the compression of the superior vena cava, thereby alleviating the patient’s clinical symptoms.

In recent years, the treatment of malignant superior vena cava obstruction by stent placement has been frequently reported. Kuo et al. pointed out that stent placement should be used as the first-line treatment for malignant superior vena cava obstruction rather than being reserved for rescue treatment after radiotherapy or chemotherapy [12]. Wei et al. believe that although stents combined with targeted drugs as secondary SVCS treatment for lung cancer cannot prolong the survival of patients, they can improve the patients’ quality of life [13].

In this study, the success rate of stent implantation technology was 100%, and all patients’ clinical symptoms were significantly relieved after surgery, which is similar to the results reported by Mokry, Maleux and Fagedet [14–16]. We believe that intolerance of surgery and a bleeding tendency are contraindications to stent implantation because anticoagulation therapy is needed after stent implantation in the superior vena cava. For patients with a bleeding tendency, anticoagulation after stent implantation may cause complications such as epistaxis, gastrointestinal hemorrhage and cerebral hemorrhage.

In this study, we observed that the restenosis rate of the superior vena cava in the Wallstent stent group (3/12) was higher than that in the ELuminexx stent group (2/18). At present, there is no definitive research showing the advantages and disadvantages of the two types of stents in the treatment of superior vena cava syndrome. However, we believe that this difference may be related to the thin silk and large mesh of the Wallstent, which easily leads to endothelialization of the stent and a relatively weak supporting force, which is one of the reasons for restenosis. Gwon et al. found that the cumulative stent patency rate of a covered stent group was significantly higher than that of the uncovered stent group, but there were no significant differences in the survival rate between the covered stent group and the uncovered stent group [17]. In this study, 2 patients underwent covered stent implantation, and there were no complications related to the stent implantation. However, covered stent graft placement may block the collateral circulation, the risk of stent displacement is higher, and the cost is higher. Therefore, we believe that their clinical use should be carefully considered according to the disease being treated.

Regarding the choice of puncture approach, puncturing the right femoral vein is more common. Because of its simple and convenient puncture, the angle from the femoral vein to the bilateral brachiocephalic vein is small, and it is easy to selectively intubate or open the superior vena cava. For cases where there is difficulty is opening the superior vena cava or they have lesions simultaneously involving the subclavian vein and internal jugular vein on the same side, the upper limb vein combined with the femoral vein two-way approach should be adopted to improve the efficiency of opening. For patients with indwelling central venous access, there is no need to repuncture without affecting subsequent operations.

In this study, 1 patient had lesions involving the right subclavian and right internal jugular veins, and the two-way approach was adopted for the right main vein and the right femoral vein to successfully open the occluded blood vessel and 2 patients were treated via the right subclavian CVC (central venous catheter) placed in the vein, so we did not have to repuncture the femoral vein.

Related literature reports that intravascular stent placement treatment has complications such as stent displacement, secondary thrombosis in the stent, restenosis, pulmonary thromboembolism, acute right heart insufficiency, and superior vena cava rupture, with an average incidence of 3.2 to 7.8% [18, 19]. Usually, superior vena cava restenosis is the most important complication because the progression of the tumor will increase the compression of the superior vena cava and even grow into the cavity through the stent network. Takahara et al. believed that even in patients with poor general conditions, additional superior vena cava stent placement is an option for the treatment of restenosis [20]. For patients with restenosis after stent implantation, balloon dilation or stent implantation can be performed at the site of stenosis to restore the reflux of the brachiocephalic vein. For tumorous lesions, reinsertion of a stent can achieve a longer-term patency rate and better clinical efficacy than balloon dilatation. Another common complication of stent implantation is secondary thrombosis in the stent. Therefore, patients in our center routinely use anticoagulant drugs after stent placement. For patients with an acute thrombosis in stents, balloon dilation and indwelling catheter thrombolytic therapy can also achieve better results. Under normal circumstances, there is no need to restent. Fagedet et al. found that stents with a diameter of more than 16 mm are more likely to experience thrombosis in the stent [16]. At the same time, a small stent diameter, long stent length, and superior vena cava endothelial injury are high-risk factors for superior vena cava restenosis. In this study, most patients were given stents with a diameter of 16 mm or less (34/37), with few complications and no serious complications related to surgery.

Although stent placement can effectively alleviate related symptoms caused by superior vena cava obstruction, follow-up treatment for the primary cause cannot be ignored. In this study, all patients received antitumor therapy after stent placement. After stent placement, active antitumor therapy, such as radiotherapy, chemotherapy and molecular targeted drugs, is an appropriate choice.

This study has certain limitations. First, this is a retrospective study with a limited number of cases. Second, due to the poor prognosis of patients with malignant tumors, the follow-up time is limited, and the long-term patency rate is unknown. Further randomized controlled experiments are needed to confirm this conclusion.

## Conclusion

In summary, percutaneous transluminal stent implantation can quickly and effectively relieve the clinical symptoms of patients with superior vena cava syndrome caused by malignant tumors, and the incidence of complications is low, so it should be regarded as a first-line treatment.

## Data Availability

The datasets used are available from the corresponding author on reasonable request.

## Abbreviations

DSA: Digital subtraction angiography
SVCS: Superior vena cava syndrome
CVC: Central venous catheter
INR: International normalized ratio
